# Neural effective connectivity explains subjective fatigue in stroke

**DOI:** 10.1093/brain/awab287

**Published:** 2021-11-17

**Authors:** Sasha Ondobaka, William De Doncker, Nick Ward, Annapoorna Kuppuswamy

**Affiliations:** 1 CoreMind Ltd, NW1 8NP London, UK; 2 Department of Clinical and Movement Neuroscience, Institute of Neurology, University College London, WC1N 3BG London, UK; 3 NHNN, University College London, WC1N 3BG London, UK

**Keywords:** poststroke fatigue, inter-hemispheric inhibition, dynamic causal modelling, paired-pulse TMS

## Abstract

Persistent fatigue is a major debilitating symptom in many psychiatric and neurological conditions, including stroke. Post-stroke fatigue has been linked to low corticomotor excitability. Yet, it remains elusive as to what the neuronal mechanisms are that underlie motor cortex excitability and chronic persistence of fatigue.

In this cross-sectional observational study, in two experiments we examined a total of 59 non-depressed stroke survivors with minimal motoric and cognitive impairments using ‘resting-state’ MRI and single- and paired-pulse transcranial magnetic stimulation.

In the first session of Experiment 1, we assessed resting motor thresholds—a typical measure of cortical excitability—by applying transcranial magnetic stimulation to the primary motor cortex (M1) and measuring motor-evoked potentials in the hand affected by stroke. In the second session, we measured their brain activity with resting-state MRI to assess effective connectivity interactions at rest. In Experiment 2 we examined effective inter-hemispheric connectivity in an independent sample of patients using paired-pulse transcranial magnetic stimulation. We also assessed the levels of non-exercise induced, persistent fatigue using Fatigue Severity Scale (FSS-7), a self-report questionnaire that has been widely applied and validated across different conditions. We used spectral dynamic causal modelling in Experiment 1 and paired-pulse transcranial magnetic stimulation in Experiment 2 to characterize how neuronal effective connectivity relates to self-reported post-stroke fatigue. In a multiple regression analysis, we used the balance in inhibitory connectivity between homologue regions in M1 as the main predictor, and have included lesioned hemisphere, resting motor threshold and levels of depression as additional predictors.

Our novel index of inter-hemispheric inhibition balance was a significant predictor of post-stroke fatigue in Experiment 1 (β = 1.524, *P* = 7.56 × 10^−5^, confidence interval: 0.921 to 2.127) and in Experiment 2 (β = 0.541, *P* = 0.049, confidence interval: 0.002 to 1.080). In Experiment 2, depression scores and corticospinal excitability, a measure associated with subjective fatigue, also significantly accounted for variability in fatigue.

We suggest that the balance in inter-hemispheric inhibitory effects between primary motor regions can explain subjective post-stroke fatigue. Findings provide novel insights into neural mechanisms that underlie persistent fatigue.

## Teaser

Persistent fatigue is a debilitating symptom in many psychiatric and neurological conditions, including stroke. Ondobaka *et al*. report that subjective poststroke fatigue can be explained by an imbalance in inter-hemispheric inhibition, revealing a new neural mechanism that could ultimately serve as a therapeutic target.

## Introduction

Fatigue is a major debilitating symptom in many psychiatric and neurological disorders,^1^ including stroke. The reported prevalence of fatigue in stroke survivors is as high as 85%, with post-stroke fatigue (PSF) having a significant impact on stroke survivors’ disability, quality of life and mortality.^2-4^ Fatigue is commonly understood as being induced by repetition of an activity, such as repeated muscle contractions. While such repetition induced muscle fatigue can be altered after a stroke, PSF refers to self-reported persistent fatigue levels unrelated to repetition induced fatigue.^5^ Such self-reported fatigue can co-occur with other affective symptoms such as depression, sleep disturbances and pain^6^^,^^7^ and less so with apathy.^8^^,^^9^ Despite such co-occurrences, PSF can occur independently and is regarded as an independent condition, a detailed discussion of which can be found elsewhere^10^. Several phenomena originating both in the central and peripheral nervous system have been suggested to play a role in development and persistence of PSF.^11–13^ Peripherally, high levels of tissue inflammation early after stroke is linked with a subsequent genesis of PSF.^14–16^ Centrally, reduced motor cortex excitability measured with transcranial magnetic stimulation (TMS),^17^ reduced inhibition of premovement inhibition^18^ and poor attention^19^ have been associated with persistent PSF. Further support for CNS involvement in fatigue comes from studies demonstrating functionally impaired motor areas involved in movement preparation,^20^ abnormal functional connectivity^21^ and altered decision making in Parkinson’s disease fatigue.^22^ Yet, the neurophysiological mechanisms that underlie persistence of PSF remain elusive.^23^

We recently hypothesized that features associated with PSF, such as reduced cortical excitability, reduced self-selected movement speeds and increased limb heaviness, could represent a deficit in sensory attenuation (i.e. inability to withdraw attention from a sensory stream).^24^ Neural control of attention, which selectively enhances and attenuates different sensory inputs, depends on the largely inhibitory inter-hemispheric connectivity in the parietal and frontal lobes.^25–28^ Inter-hemispheric inhibition is typically measured with paired-pulse TMS by comparing the amplitude of a motor-evoked potential (MEP) elicited by stimulation of the primary motor cortex (M1) with or without preceded subthreshold stimulation of the contralateral M1. To understand mechanisms that underlie PSF, here we test whether variability in self-reported PSF is associated with inter-hemispheric effective connectivity. In two separate experiments, we used computational modelling of resting-state functional MRI (rs-fMRI) and paired-pulse TMS to characterize how inter-hemispheric effective connectivity relates to PSF.

Experiments using paired-pulse TMS protocols^29^^,^^30^ provide causal evidence that transcallosal connectivity underpins control of attention and corticospinal excitability.^27^^,^^29–34^ Inter-hemispheric inhibitory effects in the normal functioning brain are not balanced between hemispheres, but exhibit an asymmetry characterized by a net left hemispheric inhibitory dominance in motor cortices^35–37^ and outside primary motor areas. ^27^^,^^34^ Inter-hemispheric inhibitory balance (IIB) can be quantified using paired-pulse TMS^35^ or effective connectivity measures derived from neuroimaging (see Friston *et al*.^38^), by subtracting the inhibitory effects of the two hemispheres from each other [(left to right) − (right to left)]. Inter-hemispheric inhibitory effects are mediated by predominantly GABAergic neurons that connect homologue regions in the two hemispheres.^39^ Spectral dynamic causal modelling (DCM) provides a model-based approach to assess correlations between blood oxygen level-dependent (BOLD) signal intensities in different regions.^38^^,^^40^ Compared to the classic functional connectivity methods,^41^^,^^42^ both spectral DCM and paired-pulse TMS offer complementary methods that can discern the directionality and the valance (net inhibition versus excitation) of the underlying neural effects.

Disturbed IIB balance is observed in various neurological and psychiatric disorders.^43–51^ For example, clinical depression is characterized by a shift in inhibitory dominance away from the left and towards right-hemisphere dominance,^48^ and excitatory repetitive TMS protocols to left frontal cortex are used to reverse the dominance to significantly decrease depression severity.^52^ In the current study, we reasoned that the nature of inter-hemispheric inhibitory effects could provide a mechanistic explanation for inter-individual variability in severity of PSF. To exclude that depression would compromise statistical inference, we included only non-depressed stroke survivors and, in our analyses, we accounted for variability in depression symptoms. Our main aim was to examine the relationship between IIB of homologue neural populations and subjectively reported PSF severity. Given IIB was previously linked with attentional and affective disorders, we hypothesized that the deviation from the naturally occurring left-hemisphere inhibitory dominance would be positively associated with the severity of persistent self-reported PSF. To test this hypothesis in non-depressed stroke survivors with minimal cognitive and motor impairment, in two separate experiments we used spectral DCM of spontaneous rs-fMRI signals and paired-pulse TMS.

## Materials and methods

### Participants

This is a cross-sectional observational study approved by the Riverside Research Ethics Committee (12/LO/1474) and the London Bromley Research Ethics Committee (16/LO/0714). All stroke survivors provided written informed consent in accordance with the Declaration of Helsinki.

#### Inclusion criteria

The diagnostic inclusion criteria for recruitment included a clinical diagnosis of a first-time ischaemic or haemorrhagic lesion, date of stroke at least 3 months from the day of testing and age ≥18 years.

#### Exclusion criteria

To avoid potential sources of bias, the following exclusion criteria were adopted: use of centrally acting medication, no contraindications to TMS and functional MRI procedures, depression scores ≥11 assessed using the Hospital Anxiety and Depression Scale (HADS) and poor function. Functional screening included upper limb functional tests and cognitive tests. Poor upper limb function was defined as having <60% of the unaffected limb score in >1 of the following measures: (i) Nine-Hole Peg Test (9HPT) to measure finger dexterity; (ii) Action Research Arm Test (ARAT); and (iii) grip strength.

### Recruitment

#### Experiment 1 recruitment: resting state functional MRI and transcranial magnetic stimulation

Stroke survivors were recruited from the Thames Stroke Research Network from the University College NHS Trust Hospital, Epsom NHS Trust Hospital, Royal Surrey NHS Trust Hospital and from community stroke groups between February 2013 and September 2014. A total of 225 stroke survivors were screened on the above-mentioned eligibility criteria. Eligibility criteria were met by 78 participants, of which we recruited 70 for the TMS session (reported in Kuppuswamy *et al*.^17^). Of the 70 stroke survivors, 18 additionally took part in the rs-fMRI scanning session and were included in this study, so their data were used for the reported analyses. There was one participant with a missing date of birth. The sample consisted of 18 (two female) non-depressed, ischaemic or haemorrhagic stroke survivors with a mean age of 58.68 ± 10.30 (mean ± standard deviation, SD), tested 4.03 ± 3.97 (mean ± SD) years post-stroke.

#### Experiment 2 recruitment: paired-pulse transcranial magnetic stimulation

Stroke survivors were recruited via the Clinical Research Network from the University College NHS Trust Hospital, departmental Stroke Database and the community between October 2017 and June 2019. A total of 132 stroke survivors were screened on the above-mentioned eligibility criteria. Eligibility criteria were met by 113 stroke survivors, of which 41 were recruited for the paired-pulse TMS experiment. The paired-pulse TMS sample consisted of 41 (nine females) non-depressed, ischaemic or haemorrhagic stroke survivors with a mean age of 62.37 ± 12.63 (mean ± SD), tested 5.46 ± 5.76 (mean ± SD) years post-stroke.

#### Fatigue and depression measurements

In both experiments, we measured fatigue using the Fatigue Severity Scale (FSS-7), a widely used and validated self-report questionnaire. FSS responses range from 1 to 7, with an average score of 7 being the highest fatigue and a score of 1 reflecting no fatigue whatsoever.^9^ We measured depression with the HADS, which is typically used in a clinical setting to assess anxiety and depression in patients with physical symptoms. With a maximum score of 21, normal scores range from 1 to 7, 8–10 are borderline cases, and 11 and above are abnormal or depressed cases.

### Experiment 1 methods: resting state functional MRI and transcranial magnetic stimulation

In two experimental sessions, we measured low-frequency spontaneous fluctuations in the rs-fMRI signal and resting motor thresholds (RMTs), a typical measure of corticospinal excitability. Our use of RMTs was motivated by its association with fatigue in our previous work. For full details of the TMS procedure used in the first session refer to Kuppuwamy *et al*.^17^ In the second session, participants underwent an eyes-open 12-min rs-fMRI using a standard scanning protocol. Scanning was performed at the Wellcome Centre for Human Neuroimaging using a 3 T Trio scanner (Siemens). All participants underwent a single scanning session during which a T_2_*-weighted MRI transverse echo-planar images were acquired using a 12-channel head coil. The resting block comprised 200 volumes of 32 slices, with a 30 ms echo time and a repetition time of 2.175 s (4.5 × 4.5 × 4.5 mm voxels). Participants were instructed to lie within the scanner, to keep their eyes closed, to remain awake and to restrict their movement as much as possible until further instructed. A high resolution T_1_-weighted anatomical images (1.3 × 1.3 × 1.3 mm voxels; 176 partitions, field of view = 256 × 240, echo time = 2.48 ms, repetition time = 7.92 ms, flip angle = 16°) and a field map (echo time 1 = 10 ms and echo time 2 = 12.46 ms, 3 × 3 × 3 mm resolution, 1 mm gap) were also acquired. This was used to create B0 field maps used by the SPM FieldMap Toolbox to unwarp the functional images.

#### Functional MRI preprocessing

We performed conventional functional imaging preprocessing using SPM12 (www.fil.ion.ucl.ac.uk/spm), including the removal of the first four volumes, realignment, spatial normalization with 3 mm cubic voxels, a spatial smoothing of 6 mm full-width at half-maximum and nuisance variable regression. The nuisance regressors included 18 motion parameters (six head motion parameters and their first and second derivatives) and the average signal strength extracted from 6 mm spheres from two (CSF and white matter) reference regions with the following Montreal Neurological Institute (MNI) (*x*, *y*, *z*) coordinates: 19, −34, 18 and 27, −18, 32. This set of nuisance variables incidentally removes low-frequency fluctuations normally associated with global confounds.

#### Dynamic causal modelling of effective connectivity

Spectral DCM provides a model-based approach to understand correlations between BOLD signal in different brain regions. Compared to the classic functional connectivity methods that mainly describe correlations between signal intensities in different regions,^41^^,^^42^ Spectral DCM offers a possibility to discern the directionality and the valance (net inhibition versus excitation) of the underlying neural influences.^38^^,^^40^ Spectral DCM models extrinsic (i.e. between regions) and intrinsic (i.e. within region) effective connectivity in the selected brain regions of interest based on the estimated cross-spectra (cross-covariances in the frequency domain) of the extracted BOLD time series. An advantage of using a model-based approach such as DCM is the opportunity to infer the biologically plausible inter-hemispheric neural interactions that underlie fatigue, compared to investigating the dynamics in the measured EEG or functional MRI signals. Examination of the model parameter values that provide best fit for the observed functional MRI signals allow characterization of the biologically plausible inter-hemispheric connectivity patterns that best explain reported fatigue.

**Figure 1:**
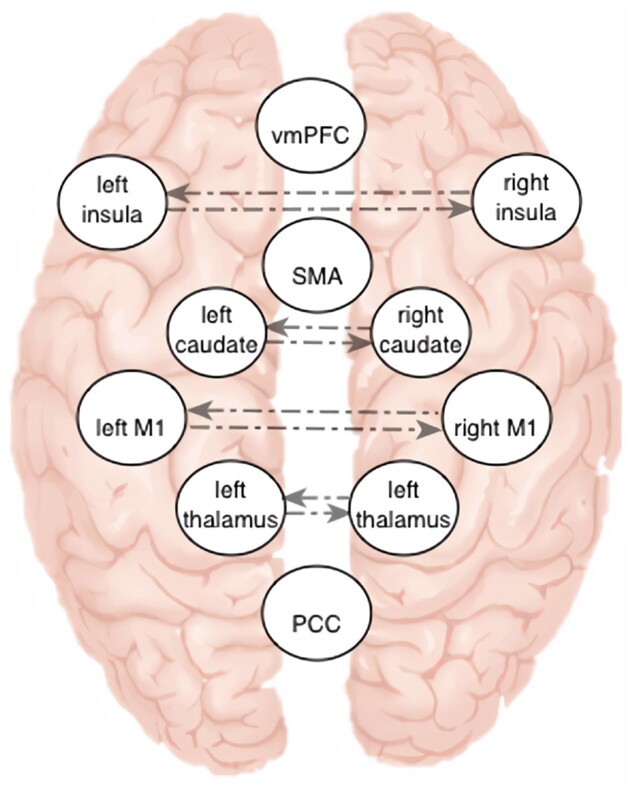
**Neural connectivity computational model architecture.** This figure represents the DCM architecture consisting of 11 brain regions selected from NeuroSynth tool [http://neurosynth.org/; ventromedial prefrontal cortex (vmPFC), left and right anterior insula, supplementary motor area (SMA), left and right caudate head, left and right primary motor cortex (M1), left and right thalamus and posterior cingulate cortex (PCC)]. The model was fully connected, consisting of 110 extrinsic (between regions) influences, and 11 intrinsic (within region) influences. Depicted are the eight inter-hemispheric influences, other connections are left out from the figure for clarity. We used this biologically plausible DCM to find the best fit or explain the recorded fluctuations in BOLD intensities.

To use spectral DCM, we extracted rs-fMRI BOLD time series from bilateral primary motor cortices (M1; 30, −24, 64; −30, −20, 66), anterior insular cortices (38, 16, 2; −36, 16, 0), thalamus (12, −12, 10; −12, −12, 10) and caudate nuclei heads (14, 12, 14; −14, 12, 14). Additionally, we included the three cortical midline regions of interest: the supplementary motor area (0, −6, 58) and the two key default mode regions, ventromedial prefrontal cortex (0, 50, −4) and posterior cingulate cortex (0, −52, 24). Inclusion of midline regions was a pragmatic choice that was expected to result in a model that better captures global endogenous neural fluctuations. Exact locations of the centres of the 8 mm region of interest spheres, indicated previously in the MNI coordinates, were determined by using NeuroSynth meta-analysis maps. NeuroSynth (http://neurosynth.org) is an online platform for large-scale automated meta-analysis of published neuroimaging results that provides posterior probabilities of a specific term being used in the abstract of the analysed publications (e.g. primary motor cortex) conditional on the presence of activation in a chosen voxel. We selected the voxels with the highest posterior probability of being associated with the terms of the 11 chosen region of interest included to capture the global neural fluctuations of both hemispheres (Fig. 1). Visual inspection of structural images showed no apparent lesions in any of the selected region of interest. The resulting specified model consisted of a fully connected architecture with 110 extrinsic connections and 11 intrinsic connections (Fig. 1).

#### Participant-specific Bayesian estimation of effective connectivity parameters

At the participant-specific level of analysis, we estimated the strength of all effective connectivity parameters for each participant using a standard Bayesian inversion scheme (Variational Laplace^53^). We used a parametric empirical (PEB, using spm_dcm_peb_fit in SPM 12) model that furnishes a more efficient and robust estimation of effective connectivity parameters by using a group mean as the empirical prior.^54^ During the iterative model estimation (i.e. Bayesian model inversion) procedure, the connectivity parameter strengths representing biologically plausible extrinsic and intrinsic effects are optimized to generate cross spectral density (CSD) so as to fit this CSD to the measured CSD (which is generated from the measured BOLD data). DCM uses negative variational free energy to approximate the log-evidence that a particular model of connectivity patterns fits or explains the observed fluctuations in the region-specific BOLD time series. In DCM, estimated positive values of extrinsic parameters represent excitatory effect and negative values index inhibitory effect from region A to region B, while their absolute values represent the percentage estimated activity change of this effect (effect size). The participants’ specific estimates were used to compute an index of inter-hemispheric connectivity used in subsequent group-level analysis (see Table 1 for the M1 summary).

**Table 1:** Effective connectivity summary

Participant	Effect size (Hz) Left to Right M1	Effect size (Hz) Right to Left M1	IHI index
Pp1	0.274	−0.347	0.621
Pp2	0.935	0.430	0.505
Pp3	−0.242	0.196	−0.438
Pp4	−0.616	−0.047	−0.569
Pp5	−0.589	0.547	−1.136
Pp6	0.006	−0.021	0.027
Pp7	0.154	−0.165	0.319
Pp8	0.134	0.061	0.073
Pp9	0.303	0.046	0.257
Pp10	0.137	0.127	0.010
Pp11	−0.058	−0.540	0.482
Pp12	−0.350	0.241	−0.591
Pp13	0.343	1.289	−0.946
Pp14	−0.329	0.341	−0.670
Pp15	0.131	−0.327	0.458
Pp16	0.189	−0.249	0.438
Pp17	0.148	−0.286	0.434
Pp18	−0.335	0.237	−0.572

Table shows a summary of the estimated M1 inter-hemispheric effective connectivity effect sizes and the computed inter-hemispheric inhibition (IHI) from Experiment 1. Pp = participant.

Using participant-specific posterior expectations for each effective connection, we computed the IIB index for the primary motor cortex, the anterior insular cortex, caudate and the thalamus by subtracting right-to-left hemisphere parameter values from the left-to-right parameter values [IIB = (left to right) − (right to left)]. The IIB index characterizes the nature of normally occurring IIB in the individual ‘resting’ brain. Negative IIB values reflect a stronger left-to-right net inhibitory effect, whereas positive values reflect a stronger inhibitory right-to-left effect.

#### Group-level multiple regression of Fatigue Severity Scale values from the inhibitory balance indices

Finally, at the between-participants (group) level analysis, to explain individual differences in subjectively reported FSS (fatigue) scores we used the participant-specific IIB indices and the interaction between lesioned hemisphere (left versus right) and IIB indices. The interaction term was included to test for a potential confounding effect of the lesioned hemisphere. We used classic multiple linear regression analysis in R software (RStudio v.1.2.5033) to test whether the explanatory variables significantly explain variance in the FSS target variable. We applied the Bonferroni correction to a chosen significance threshold of *P* < 0.05 to control for false positives over four tests of IIB in M1, insula, caudate and the thalamus. We used Hotelling’s *t*-test to examine whether there were statistical differences in explanatory power of the four IIB indices, i.e. to test for the interaction in the strength of associations. In a second multiple regression model, we tested whether IIB uniquely explains FSS when HADS depression scores and individual RMTs are added as independent explanatory variables. In a third and final model, we also included age and sex as independent explanatory variables to control for their influence. The best fitting model was determined using the Bayesian Information Criterion (BIC), with a lower BIC indicating a better fitting model. Assumptions of normality and homoscedasticity of the residuals for each linear regression model were assessed visually using quantile–quantile normal plots and fitted- versus residual-value plots.

### Experiment 2 methods: paired-pulse transcranial magnetic stimulation

In Experiment 2, we used paired-pulse TMS to quantify the extent of inter-hemispheric inhibition between the homologue primary motor cortices. Experiment 2 was motivated by the findings from Experiment 1 and has aimed to provide an inter-methodological cross-validation using paired-pulse TMS. A conditioning TMS pulse (CP) was applied to the M1 on one hemisphere, followed 10 ms later by a test TMS pulse (TP) delivered to the M1 of the other hemisphere. Trials using double pulses (CP–TP) were randomly intermixed with those containing test pulse alone, with 20 trials for each condition, giving a total of 40 trials. The intertrial interval was set to 7 s (±1.4 s). Inter-hemispheric inhibition was calculated as the amplitude of the conditioned MEP in the double-pulse trials (CP–TP) relative to the amplitude of the test MEP when the test pulse was delivered alone: TP–(CP–TP). This process was then repeated for the other hemisphere, giving us a measure of inter-hemispheric inhibition from the left-to-right hemisphere (IHI_LtoR_: conditioning pulse applied to the left hemisphere and test pulse applied to the right hemisphere) and from the right-to-left hemisphere (IHI_RtoL_: conditioning pulse applied to the right hemisphere and test pulse applied to the left hemisphere).

#### Surface EMG and transcranial magnetic stimulation

EMG recordings were carried out on the first dorsal interosseous muscle of both hands using neonatal prewired disposable electrodes (1041PTS Neonatal Electrode, Kendell) in a belly-tendon montage. The ground electrode was positioned over the flexor retinaculum of the hand. The signal was band pass filtered (20–1000 Hz), amplified with a gain of 1000 (D360, Digitimer), digitized at 5 kHz (Power1401, CED) and recorded with Signal v.6.04 software (CED). TMS was delivered using two magnetic stimulators (Magstim 200^2^, Magstim), each connected to a figure-of-eight coil (70 mm diameter for the test pulse and 50 mm diameter for the conditioning pulse). The magnetic coil for the test pulse was held tangentially on the scalp at an angle of 45° to the mid-sagittal plane to induce a posterior-anterior current across the central sulcus. The magnetic coil for the conditioning pulse was held at an angle of 90° to the mid-sagittal plane. The participants were instructed to stay relaxed with their eyes open and their legs uncrossed. The motor ‘hotspot’ of the first dorsal interosseous muscle for each hemisphere was determined as previously.^17^ Both coils were held at the hotspot of the first dorsal interosseous muscle in each hemisphere during the stimulation protocol. The stimulator setting for both stimulators was adjusted to produce a target MEP size of 0.5 mV. This was defined as the stimulator setting (determined to the nearest 1% of maximum stimulator output) required to evoke a peak-to-peak MEP amplitude of ≥0.5 mV in a minimum of 5 of 10 consecutive trials.

#### Transcranial magnetic stimulation data analysis

The data files were extracted from Signal into MATLAB and were analysed offline using custom-written routines in MATLAB (2018a, MathWorks). MEP peak-to-peak amplitude was measured on a trial-by-trial basis from the acquired EMG signal without applying any additional filters. Resting EMG was defined as the root mean square across all trials for each participant in the 100 ms preceding the TMS pulse of each trial. Thresholds set at four times these levels were used to identify any muscle contraction preceding the stimulation. All trials were then visually inspected to ensure that no build-up of EMG was apparent before the TMS. Trials containing outlier MEP amplitudes (Grubb’s test, *P* < 0.005) were also excluded from the final analysis. On average, 7.9% of TMS trials were excluded across all stroke survivors with a minimum of 15 trials per condition.

Identical to the procedure in Experiment 1, the IIB index was computed by subtracting the right-to-left inter-hemispheric inhibition from the left-to-right inter-hemispheric inhibition (IIB = IHI_LtoR_ − IHI_RtoL_). IIB index characterizes the naturally occurring IIB in the individual ‘resting’ brain. Negative IIB values reflect stronger left-to-right inhibition, whereas positive values reflect stronger right-to-left inhibition.

#### Group-level multiple regression of Fatigue Severity Scale values from the inhibitory balance indices

To explain individual differences in subjectively reported FSS-7 (fatigue) scores, we have again implemented a multiple linear regression in R (RStudio v.1.2.5033). We used the same full model as in Experiment 1 including IIB and the interaction between lesioned hemisphere and IIB, HADS depression scores and RMTs. Assumptions of normality and homoscedasticity of the residuals were assessed visually using quantile–quantile normal plots and fitted- versus residual-value plots.

### Data availability

Raw data can be made available on request.

## Results

### Participants’ characteristics

In Experiment 1, six stroke survivors had a right-hemisphere stroke and 11 had a left-hemisphere stroke (Table 2). Participant characteristics indicated low cognitive impairment, indexed by unaffected mental speed [Symbol Digit Modalities Test (SDMT) scores of 1.25 ± 0.42]. Participants also showed low motoric impairment, reflected in the measured 9HPT (77.73 ± 33.06%), ARAT scores (96.01 ± 11.17%) and preserved grip strength (88.67 ± 22.14%) of the unaffected side. In Experiment 1, ischaemic strokes were predominantly lacunar (localized and small in size), whereas the three haemorrhagic strokes were associated with an extensive cortical damage. We ensured that none of the 11 region of interest used to build our DCM model had any noticeable structural damage.

**Table 2:** Stroke summary

Participant	Hemisphere	Location	Stroke type
Pp1	Left	Parietal cortex	Ischaemic
Pp2	Left	Putamen	Ischaemic
Pp3	Right	Parietal cortex	Ischaemic
Pp4	Left	Prefrontal cortex	Ischaemic
Pp5	Right	External capsule	Ischaemic
Pp6	Left	Temporal cortex	Ischaemic
Pp7	Right	Corona radiata	Ischaemic
Pp8	Left	Prefrontal cortex	Ischaemic
Pp9	Right	Internal capsule	Ischaemic
Pp10	Right	Parietal cortex	Ischaemic
Pp11	Left	Parietal cortex	Haemorrhagic
Pp12	Left	Parietal cortex	Haemorrhagic
Pp13	Left	Putamen	Ischaemic
Pp14	Left	External capsule	Ischaemic
Pp15	Left	Pons	Ischaemic
Pp16	Left	Fronto-parietal	Haemorrhagic
Pp17	Right	Putamen	Ischaemic
Pp18	Left	Insula	Ischaemic

Table shows a summary of the stroke-affected hemisphere, locations and type of the stroke from Experiment 1. Pp = participant.

In Experiment 2, 20 stroke survivors had a left-hemisphere stroke and 21 had a right-hemisphere stroke. Participant characteristics indicated low cognitive impairment, indexed by unaffected mental speed (SDMT scores of 1.18 ± 0.46). Participants also showed low motoric impairment, reflected in the measured 9HPT (89.31 ± 22.38%), ARAT scores (99.60 ± 1.56%) and preserved grip strength (95.55 ± 15.45%) of the unaffected side. MRI data were not consistently available for the participants in Experiment 2. Participants in the two experiments did not differ in any of the relevant indices (Table 3).

**Table 3:** Participants’ demographics

	Experiment 1	Experiment 2	*P*-value
	(*n* = 18)	(*n* = 41)	
Gender, females: males	2:16	9:32	0.4756
Hemisphere affected, left: right	12:6	20:21	0.4122
Age, years	58.68 (10.30)	62.37 (12.63)	0.1691
Time since stroke, years	4.03 (3.97)	5.46 (5.76)	0.2416
Grip, % unaffected hand	88.67 (22.14)	95.55 (15.45)	0.3471
NHPT, % unaffected hand	77.73 (33.06)	89.31 (22.38)	0.3066
SDMT	1.25 (0.42)	1.18 (0.46)	0.6187
Fatigue (FSS)	3.71 (1.87)	3.65 (1.98)	0.8302
Depression (HADS)	4.28 (3.27)	4.59 (3.33)	0.6020
Anxiety (HADS)	6.50 (5.45)	5.22 (3.91)	0.6083

Values are presented as mean (SD). Table presents an overview of participants’ demographics, and several measure of their physical and mental states separately for experiments 1 and 2. Last column shows *P*-values for the comparison between the experiments.

### Inter-hemispheric balance explains level of persistent fatigue

**Figure 2:**
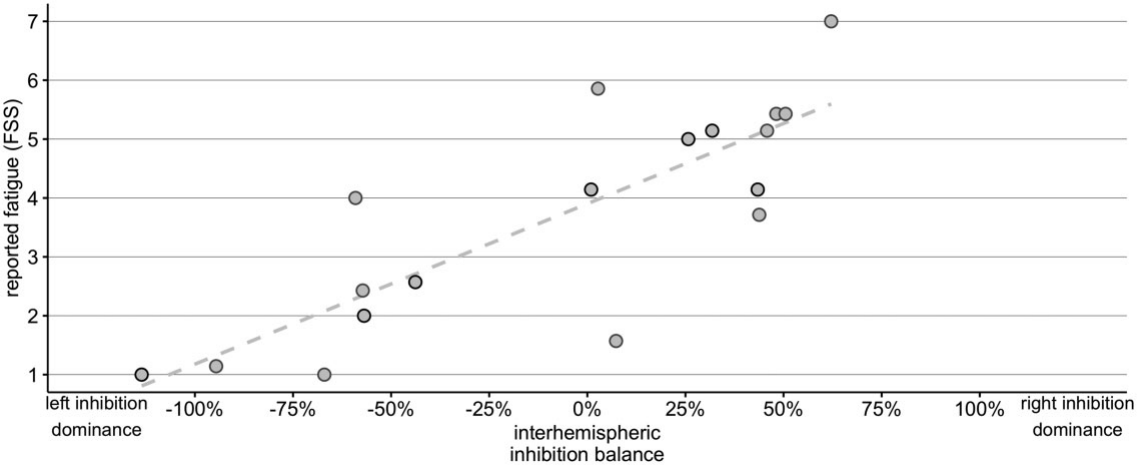
**Rs-fMRI inter-hemispheric inhibition balance in M1 and fatigue severity.** Figure shows a relationship between IIB indices (left to right − right to left influence) and self-reported fatigue severity scores in Experiment 1. The IIB was computed by subtracting right to left M1 effect sizes from left to right M1 effect sizes. Negative IIB values on the *x*-axis reflect overall stronger inhibitory left to right influence, whereas positive values reflect overall stronger inhibitory right to left influence. Strength of effective connectivity inferred from spectral DCM is represented by a percentage change in activity (effect size) in an area (e.g. right M1), as a consequence of activity change in another area (i.e. left M1). Grey circles mark participants with the left-hemisphere lesion, black circles mark participants with the right-hemisphere lesion.

The result from the multiple linear regression analysis from Experiment 1 showed that the individual IIB in the motor cortex inferred from spectral DCM explains reported levels of persistent fatigue. IIB in M1 explained reported FSS scores [β = 1.524, *P* = 7.56 × 10^−5^, confidence interval (CI): 0.921 to 2.127; Fig. 2], whereas lesioned hemisphere did not influence the IIB-FSS association (β = 0.056, *P* = 0.912, CI: −1.013 to 1.125, for IIB × Lesioned hemisphere interaction), accounting for 63% of the variability in the reported fatigue scores (adjusted *R*^2^ = 0.629). We did not observe any significant effects at the Bonferroni corrected threshold of 0.0125 when the FSS values were regressed on the IIB scores from the insula (β = −0.4850, *P* = 0.327, CI: −1.505 to 0.535), caudate (β = 0.4385, *P* = 0.3288, CI: −0.487 to 1.364) and the thalamus (β = −0.9613, *P* = 0.0415, CI: −1.881 to −0.042). Moreover, Hotelling *t*-tests revealed that FSS-M1 IIB correlation was significantly different from the correlations between FSS and insula IIB (*t* = 4.550, *P* < 0.001), caudate IIB (*t* = 3.440, *P* = 0.004) and thalamus IIB (*t* = 7.364, *P* < 0.001).

**Figure 3:**
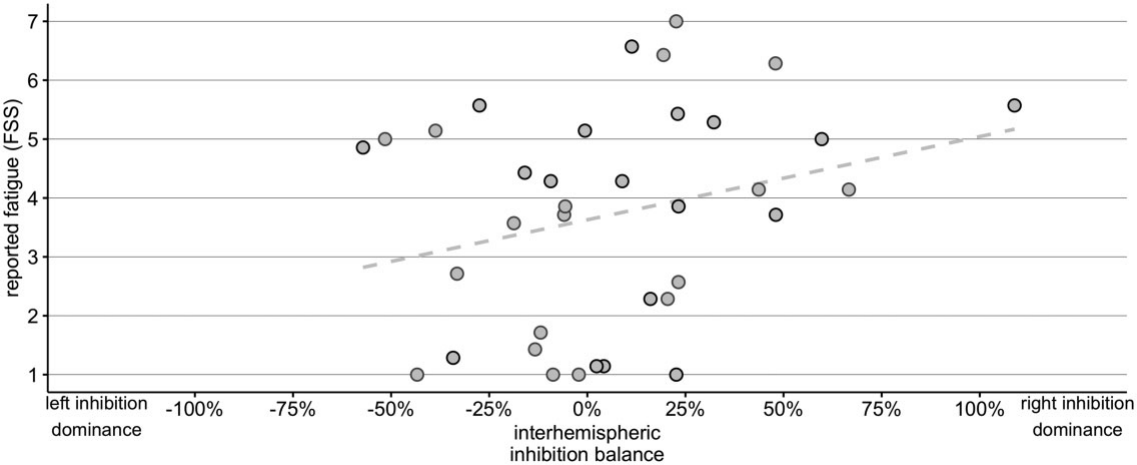
**Paired-pulse TMS inter-hemispheric inhibition balance in M1 and fatigue severity.** Figure shows a relationship between IIB indices in M1 (left to right − right to left effect) and self-reported fatigue severity scores in Experiment 2. The IIB was computed by subtracting right to left M1 effect sizes from left to right M1 effect sizes. Negative IIB values on the *x*-axis reflect overall stronger inhibitory left to right effect, whereas positive values reflect overall stronger inhibitory right to left effect. Strength of effective connectivity measured with paired-pulse TMS is represented by a percentage change in activity (effect size) in an area (e.g. right M1), as a consequence of activity change in another area (i.e. left M1). Grey circles mark participants with the left-hemisphere lesion, black circles mark participants with the right-hemisphere lesion.

Additional multiple regression analysis included individual RMTs and HADS_Depression_ scores next to IIB scores to test and account for effects of cortical excitability and depression in a single model. The results demonstrated IIB was a unique significant explanatory variable (β = 1.524, *P* = 0.026, CI: 0.163 to 2.120, BIC = 67.37) of variability in FSS fatigue scores. While the interaction between lesioned hemisphere and IIB was not a significant predictor of FSS-7 (β = −0.051, *P* = 0.924, CI: −1.177 to 1.075), nor were HADS_Depression_ and RMT (β = 0.357, *P* = 0.256, CI: −0.291 to 1.005; and β = 0.422, *P* = 0.352, CI: −0.521 to 1.364, respectively]. The multiple regression model that included participant’s age and sex as additional predictors did not significantly improve the model fit (BIC = 72.76). However, when accounting for the effect of age and sex, IIB was still a unique significant explanatory variable (β = 1.179, *P* = 0.037, CI: 0.088 to 2.270) of variability in FSS fatigue scores while none of the other additional explanatory variables were significant predictors of FSS-7.

In an independent sample of stroke survivors, in Experiment 2 we used individual IIB in the motor cortices, directly measured with paired-pulse TMS, to explain reported levels of persistent fatigue. Again, we used multiple regression analysis using the same explanatory variables as in the best fitting model in Experiment 1: lesioned hemisphere, RMTs and HADS depression scores. In line with findings from Experiment 1, results showed evidence that IIB in M1 explains individual FSS scores (β = 0.541, *P* = 0.049, CI: 0.002 to 1.080), while lesioned hemisphere interaction with IIB was not a significant predictor (β = −0.122, *P* = 0.874, CI: −1.682 to 1.437). Next to IIB, HADS depression score (β = 0.981, *P* = 0.001, CI: 0.419 to 1.543) and RMTs (β = 0.542, *P* = 0.043, CI: 0.018 to 1.066) were also independent significant predictors of FSS. Stroke survivors with low persistent PSF had low corticospinal excitability and low self-reported depression scores; survivors with high fatigue had relatively high corticospinal excitability and high depression scores.

## Discussion

Optimal IIB is fundamental for healthy neurological and psychological functions. Our results show an association between individuals’ levels of IIB in primary motor cortices (M1) and their reported levels of persistent PSF. These findings were replicated in two independent experiments using complementary neuroscientific methodology—computational modelling of rs-fMRI signals and paired-pulse TMS measuring inter-hemispheric inhibition. Next, we discuss our findings in light of previous work and theorize about the functional and biological mechanisms that underlie inter-hemispheric balance.

### Inter-hemispheric inhibitory balance and post-stroke fatigue

Healthy brains exhibit a left-dominant IIB. We showed enhanced right-hemisphere inter-hemispheric inhibitory dominance is related to the experience of high PSF. Higher left-hemisphere inter-hemispheric inhibitory dominance was associated with low PSF, with IIB explaining a large proportion of inter-subject variability in measured fatigue severity. Our results are in keeping with findings that link chronic fatigue in multiple sclerosis to inter-hemispheric balance disturbances indexed by the measured EEG activity.^21^ This indicates that similar biological mechanisms might be at the base of persistent fatigue state, irrespective of whether it was initially triggered by a stroke or multiple sclerosis.^24^ Generally, the opposite pattern of inter-hemispheric connectivity in PSF is in agreement with the disturbed patterns of inter-hemispheric connectivity associated with many neurological and psychiatric disorders.^43^^,^^44^^,^^46–51^

Particularly relevant is the agreement with the typically observed right-hemisphere inhibitory dominance and the elevated corticospinal excitability in clinical depression,^48^ a multifaceted disease that includes fatigue as a principal symptom. Our results from the paired-pulse TMS experiment that reveal an association between self-reported depression and fatigue are in congruence with the view in which depression and persistent fatigue partly share a common underlying mechanism. Notably, overall, the studied population had an average HADS depression score of 4.3 (Experiment 1) and 4.6 (Experiment 2) out of 21 (8 being borderline and 11 abnormal threshold), making depression an unlikely confound. Subjective fatigue measured by FSS-7 is shown to be unrelated to apathy, a syndrome that could be confused with fatigue. The original FSS-9 scale included an item assessing motivation that is now excluded due to its repeated inconsistency with fatigue, rendering the confusion unlikely (see Johansson *et al*.^9^). The affected hemisphere did not influence our findings as indicated by the absence of its significant interaction with IIB.

### Post-stroke fatigue and cortical excitability

Previously, we showed an association between self-reported fatigue and corticospinal excitability in a sample of 70 patients with PSF.^17^ The current finding of a negative association between corticospinal excitability and PSF from Experiment 2 is well in line with the main findings from our previous work. Whereas cortical excitability and IIB were both independent explanations for fatigue in Experiment 2, IIB was a sole significant predictor of fatigue in Experiment 1, over and above the contributions of cortical excitability that showed similar non-significant pattern. Distinct results associating both IIB and cortical excitability to PSF beg the question how we can synthesize the effects of these two physiological measures on fatigue. One simple hypothesis that could provide a synthesis of these results is that resting cortical excitability causally depends on inter-hemispheric connectivity. This view is congruous with data from repetitive TMS work showing that inhibitory stimulation to M1 effects cortical excitability in the contralateral M1.^32^^,^^33^ Similarly, changes in cortical excitability in one M1 elicited by stimulating homologue area in the other hemisphere are dependent on transcallosal connectivity.^44^ Dependency of cortical excitability on intra- and inter-hemispheric cortical connectivity emphasizes crucial importance to increase understanding how wider network effect local excitation-inhibition dynamics. Although, the M1–IIB effect differed significantly from the other examined IIB effects, we do not provide evidence that M1–IIB is an exclusive explanation for PSF. Enhanced knowledge of causal network interactions using effective connectivity methods could improve future treatments aiming to modulate IIB to ameliorate PSF and related affective disorders.

### Functional and neural mechanisms of inter-hemispheric inhibitory balance

A fundamental question is what are the functional and biological mechanisms that underlie inhibitory inter-hemispheric balance? One proposal links variation in intra- and inter-hemispheric effects to asymmetry in the autonomic nervous system activity.^55^ Craig^55^ proposes that the left hemisphere shows a stronger association with processing of parasympathetic information and the right hemisphere with processing of sympathetic information. Perhaps, the inhibitory dominance of the left hemisphere over the right hemisphere as shown by TMS inter-hemispheric protocols reflects, or is even driven by, the balance of autonomic functions of the two hemispheres. The shift in balance to right-hemisphere dominance (sympathetic dominance) could also explain the inability to attend away from current sensory inputs (poor sensory attenuation). Recent work proposed poor sensory attenuation of incoming sensory information as a potential mechanism underpinning PSF.^24^ Further research is necessary to understand the role of autonomic activity in inter-hemispheric effects and consequently its effect on PSF and other affective disorders.

### Limitations

The exclusion of stroke survivors with high levels of depression, motoric and cognitive impairments could limit generalizability of our findings. In this study, stroke survivors with high levels of depression were excluded to isolate the group with fatigue without psychiatric comorbidities, as clinical depression has already been associated with aberrant inter-hemispheric connectivity.^48^^,^^52^ The exclusion of stroke survivors with depression has not introduced any potential bias to the interpretations of the data as the focus of the study was to understand biological mechanisms of PSF. To account for the relationship between depression and fatigue, measures of depressive symptoms were included in the analysis. The study did not include explicit measures of apathy, a motivational syndrome that shares attributes with persistent fatigue. However, fatigue, as measured by FSS-7, has been shown to be extraneous to motivation and apathy making it unlikely to compromise content validity of our fatigue measure.^9^ We have also excluded participants with large motoric and cognitive impairments, a pragmatic choice that should not lead to limited generalizability of our findings to fatigued stroke survivors with larger impairments.

### Future directions

The strong explanatory power of resting inter-hemispheric inhibitory connectivity makes it a potential target for new PSF intervention protocols using transcranial magnetic or electric stimulation. More specifically, brain stimulation methods aiming to recover physiological IIB and optimal cortical excitability could be used to ameliorate fatigue symptoms.^52^ An advantage of using a model-based approach such as dynamic causal modelling is the opportunity to infer the biologically plausible inter-hemispheric neural connectivity that underlies fatigue, compared to investigating the dynamics in the measured EEG or functional MRI signals. Examination of the model parameter values that provide best fit for the observed functional MRI signals allow characterization of the biologically plausible directionality and the sign of the inter-hemispheric connectivity patterns that best explain reported fatigue. A promising avenue for the future is to combine patient specific characterization of inter-hemispheric balance, inferred from TMS, functional MRI or EEG signals, with neuro-stimulation protocols that apply TMS, DC or AC current to rebalance the disturbed inter-hemispheric dynamics. A first step on this avenue would be to better understand the autonomic and CNS mechanisms behind the reversal of inter-hemispheric dominance.

## Conclusion

We suggest that the balance in inter-hemispheric inhibitory connectivity between primary motor regions is involved in persistence of subjective fatigue. Our findings that PSF is associated with altered effective connectivity in M1 support the importance of optimal IIB for healthy brain functioning.

## Abbreviations

BOLD: blood oxygen level-dependent
DCM: dynamic causal modelling
FSS-7: Fatigue Severity Scale
HADS: Hospital Anxiety and Depression Scale
IIB: inter-hemispheric inhibitory balance
M1: primary motor cortex
MEP: motor-evoked potential
PSF: post-stroke fatigue
RMT: resting motor threshold
rs-fMRI: resting-state functional MRI
TMS: transcranial magnetic stimulation
